# Gender-Affirming Hormone Therapy: Physical and Sociopsychological Effects, Impact and Satisfaction

**DOI:** 10.7759/cureus.36484

**Published:** 2023-03-21

**Authors:** Rafael B Santos, Carolina Lemos, Miguel Saraiva

**Affiliations:** 1 Endocrinology Department, Centro Hospitalar Universitário do Porto, Porto, PRT; 2 Population Studies, Instituto de Ciências Biomédicas Abel Salazar, Porto, PRT

**Keywords:** satisfaction, impact, effects, gender-affirming hormone therapy, transgender

## Abstract

Background

Gender dysphoria treatment includes gender-affirming hormone therapy (GAHT). Studies are still lacking on how to characterize its effects and impact on transgender people's lives more effectively.

Aim

To study the physical and psychological effects of GAHT on transgender individuals, assess its impact on their lives, and rate their overall satisfaction.

Methods

Participants (n = 114; ages 18-62 years; median age 24.0 (21.0 - 33.0) years) included transgender adults residing in Portugal who were undergoing or had undergone hormonal therapy for at least one uninterrupted year. Participants completed an original questionnaire. For most items, an ordinal Likert-style scale ranging from 0 (worst result) to 6 (best result) was used. Descriptive statistics and non-parametric tests, including Pearson's chi-squared test, Wilcoxon signed-rank test, and Mann-Whitney U test were used to analyze categorical and continuous variables, with a significance level set at 0.05.

Outcomes

The outcomes included desired physical changes rating (perception and satisfaction with changes); side effects of GAHT; the sociopsychological impact of GAHT (on self-esteem, body image, psychological wellbeing, social and family relations); overall satisfaction (with treatment results and medical follow-up).

Results

The changes classified as the most perceptible in those undergoing masculinizing treatment (Group M) were amenorrhea (6 (5.0-6.0) points) and clitoris enlargement (6 (5.0-6.0) points). These were also the ones rated as the most satisfactory (6 (6.0-6.0) points for amenorrhea and 6 (4.0-6.0) points for clitoris enlargement). On those undergoing feminizing therapy (Group F), the alteration voted as the most perceptible was sperm production decrease (6 (2.0-6.0) points), and the ones classified as the most satisfactory were sperm production decrease (6 (4.0-6.0) points) and spontaneous erections decrease (6 (5.0-6.0) points). Side effects were reported by 89.7% of Group M (mood swings were the most common) and 96.3% of Group F* *(decreased libido was the most frequent). The sociopsychological impact of hormonal treatment was significantly positive in all analyzed variables (p<0.001). Overall satisfaction with treatment results and medical follow-up were rated with 5 points and 4.5 points, respectively.

Clinical implications

This study provides clinicians with more evidence that GAHT may improve the physical, psychological and social health of transgender people seeking medical transition.

Strengths and limitations

The strengths of the current study include a high participant count relative to the target population, the acquisition of data on previously unexplored variables, and the significance of being one of the few investigations of its kind conducted in Portugal. However, the study has limitations, including differences in participant characteristics, a small sample size for some variables, potential bias due to the retrospective nature of the study, individualized treatment regimens, and the inclusion of participants from different countries, which limit the generalization of the results.

Conclusions

This study provides further evidence that GAHT is effective, and that its physical effects are satisfactory while resulting in mostly non-severe nor life-threatening side effects. GAHT is an important therapy in gender dysphoria and has consistent results in improving numerous sociopsychological variables.

## Introduction

Gender dysphoria (GD) is characterized by clinically significant distress or impairment in important areas of functioning, that can arise in many transgender people as a consequence of the incongruence between the experienced gender and the one assigned at birth [1].

In Portugal, being transgender is legal and protected under anti-discrimination laws [2]. Transgender individuals have the right to change their name and gender on legal documents, and gender-affirming healthcare is available through the public health system [2]. In the last years, there has been an increasing acceptance of gender diversity and transgender rights, and Portugal's Gender Identity Law of 2011 is a significant aspect of this change, allowing individuals to change their gender marker on identification documents without the need for medical procedures [2,3]. However, despite the legal protections in place, there is still discrimination and stigmatization of transgender people in society, particularly in relation to employment, education, and access to healthcare [2]. Although more public hospitals are offering gender-affirming treatments, there are still long waiting lists for these services, indicating the need for further improvements [4]. The prevalence of transgender individuals in Portugal is unknown, but national data shows that, between 2011 and 2021, 1227 transgender Portuguese changed their gender marker in the Civil Registration System [3].

GD treatment consists of social transition, psychotherapy, hormone therapy, and surgery [5]. Studies have shown that these therapies are effective in treating GD and can lead to improvements in mental health issues [6]. While the treatment plan is personalized, hormone therapy is often the initial medical intervention and is based on individual objectives, risk-benefit analysis, the presence of comorbidities, and socio-economic factors [5]. Masculinizing therapy is typically achieved through testosterone while feminizing hormone therapy involves a combination of estrogen and an androgen blocker, which is usually more complex [5].

The timing of appearance of the desired physical changes is highly variable: some features might take as little as one month to be noticeable, while others can take up to six or more months, and even years to reach their peak [7]. The main expected effects of masculinizing therapy are amenorrhea, clitoris enlargement, and voice deepening while feminizing therapy most markedly induces breast development, softer/less oily skin, and a decrease in semen production, spontaneous erections, and testicle size [7]. Also, masculinizing therapy is expected to increase body and facial hair, decrease body fat mass and increase lean mass, on the other hand, feminizing hormone therapy is expected to have the opposite effects [7].

Gender-affirming hormone therapy (GAHT) is not exempt from side effects and may be associated with erythrocytosis, weight gain, acne, alopecia, and sleep apnea in masculinizing therapy [5,7]. In feminizing hormone therapy, side effects include venous thromboembolism, gallstones, hepatic toxicity, weight gain, dyslipidemia, and cardiovascular disease [5,7]. 

The first aim of our study was to evaluate how significant the perception of the desired physical changes was and the consequent satisfaction with such changes after at least one year of uninterrupted GAHT in transgender individuals.

Our second aim was to explore the impact of GAHT on trans people’s lives regarding sociopsychological variables such as self-esteem, body image satisfaction, psychological well-being, and social and familial relations, by rating them with a pre- and post-therapy score.

The third and last objective was to rate participants’ overall satisfaction with both therapy results and medical follow-up during treatment.

With this study, we hope to get better enlightenment on GAHT’s benefits and risks so that physicians can provide more evidence-based care to transgender patients. It is also our desire to encourage other investigators to conduct studies in this under-researched area.

## Materials and methods

Participants and procedure

We performed a cross-sectional study in March 2021.

The participants of this study were adult transgender individuals, residing in Portugal, currently undergoing or had undergone GAHT, regardless of the timeframe in which the treatment was conducted.

Data was collected through an original non-validated questionnaire that was sent to the participants via e-mail. The authors developed the questionnaire in collaboration with Portuguese psychologists specializing in the healthcare of transgender individuals. The questionnaire was based on questions from several validated questionnaires freely available online, such as the Gender Identity/Gender Dysphoria Questionnaire for Adolescents and Adults (GIDYQ-AA), the Trans Woman Voice Questionnaire (TWVQ) and the Body Image Scale (BIS). To identify potential participants for the study, we collaborated with 12 Portuguese LGBTQI+ associations and organizations. These groups helped us by accessing their databases and sending an invitation to all their registered transgender associates, inviting them to participate in the study. The invitation contained the questionnaire Google Forms' link and all transgender associates who did GAHT were invited to participate. We obtained a total of 147 responses, of which 142 were valid: two were excluded because they were duplicated, and three were excluded because the answers weren't congruent throughout the questionnaire. 

Having the aim of this article in mind, only the answers of participants who took GAHT for at least one uninterrupted year were included (regardless of still being on GAHT or not), resulting in a total of 114 relevant answers to the questionnaire, of which 87 were from individuals on masculinizing therapy (Group M) and 27 from people undergoing feminizing treatment (Group F). All data was registered anonymously, respecting participants’ data protection.

This study was approved by the local ethics committee ("Ethics Committee CHUP/ICBAS - DEFI"). Informed consent was explained at the beginning of the questionnaire, and participants had to give consent to proceed with the questionnaire.

Measures

The study's questionnaire was created by the authors using the Google Forms platform, consisting of 60 different questions, 19 of each addressing demographics, GAHT physical and sociopsychological effects, impact, and consequent satisfaction with both results and medical follow-up. Other questions aimed to evaluate how the treatment was being oriented, participants’ knowledge about the transition process, therapy regimen safety, adhesion, and compliance. For this article's aim, only the answers to the first 19 questions are presented and discussed. 

To answer some of the items on the questionnaire, an ordinal Likert-style scale ranging from 0 (worst result) to 6 (best result) was used.

For the first aim, regarding GAHT effects, participants rated their perception of physical changes development from “0 - no changes” to “6 - very significant changes” and their satisfaction with them from “0 - not satisfied” to “6 - totally satisfied”. Participants also indicated the physical and psychological adverse effects that they experienced by choosing them from a list presenting the most common side effects, explained with common nonmedical language, while also having the option of writing down any other one not listed. 

To achieve the second goal, respondents evaluated GAHT’s sociopsychological impact, rating the following variables before and after at least one year of GAHT, in a retrospective manner: self-esteem (“0 - extremely low self-esteem” to “6 - extremely high self-esteem”), body image satisfaction (“0 - total body dissatisfaction” to “6 - total body satisfaction”), psychological wellbeing (“0 - recurrent/constant suicidal thoughts” to “6 - no suicidal thoughts”), social relations (“0 - isolation and no friends” to “6 - extremely sociable and has friends”) and family relations (“0 - awful/conflicted/nonexistent family relations” to “6 - great family environment”).

The third goal was accomplished by rating the general satisfaction with GAHT’s results and with the medical follow-up received during the treatment with a scale ranging from “0 - not satisfied” to “6 - totally satisfied”.

Statistical analysis

Categorical variables were presented as frequency distribution and compared using Pearson's chi-squared test. For each continuous variable, normality was tested by histogram observation and the Kolmogorov-Smirnov test and, since the almost totality of continuous variables had a non-normal distribution, results were presented as medians (followed by interquartile ranges). Samples were compared using nonparametric tests, such as Wilcoxon signed-rank test for paired variables and the Mann-Whitney U test for independent variables, with a significance level set at 0.05. Data analysis was performed using the SPSS Statistics v. 27 software (IBM Corp., Armonk, NY).

## Results

Demographics

**Figure 1:**
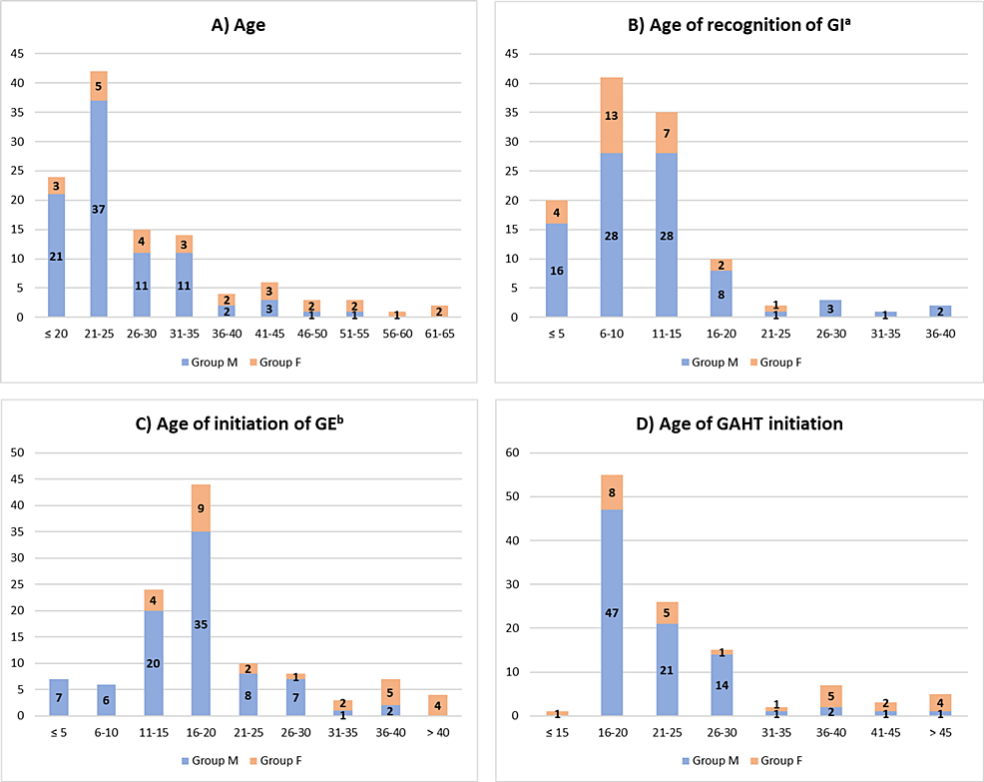
Participant demographics Results are presented as absolute frequencies ^a^ Gender identity; ^b^ gender expression Abbreviations: GAHT, gender-affirming hormone therapy

Respondents’ age ranged from 18 to 62 years and their median age was 24.0 (21.0 - 33.0) years. Participants’ median age of recognition of self-gender identity was 8.0 (8.0 - 14.0) and initiation of public expression of their experienced gender was 18.0 (14.5 - 22.5) years of age. Participants initiated hormonal therapy at a median age of 21.0 (18.0 - 27.0). Figure 1 presents this data organized in stacked bar charts.

Most participants had treatment for two to five years (40.4%; M=40.2%; F=40.7%), followed by one to two years (38.6%; M=41.4%; F=29.6%) and five years or more (21.1%; M=18.4%; F=29.6%). Table 1 presents this and other demographic information on participants.

**Table 1:** Participant demographics Results are presented as percentages (%) ^a^ This includes same-sex marriage, which is legal in Portugal since 2010

Variable	Total (N=114)	Group M (n=87)	Group F (n=27)
Place of birth			
Portugal	91.2	95.4	77.8
Brazil	4.4	2.3	11.1
Mozambique	1.8	1.1	3.7
France	0.9	1.1	
Switzerland	0.9		3.7
Venezuela	0.9		3.7
Area of residence			
North	34.2	33.3	37.0
Center	19.3	20.7	14.8
Lisbon Metropolitan Area	38.6	36.8	44.4
Alentejo	3.5	3.4	3.7
Algarve	2.6	3.4	
Azores	0.9	1.1	
Madeira	0.9	1.1	
Education			
Primary school (1st - 9th grade)	7.0	4.6	14.8
High school (10th - 12th grade)	43.9	43.7	44.4
Vocational education	14.0	13.8	14.8
Bachelor's degree	24.6	28.7	11.1
Master degree	10.5	9.2	14.8
Professional status			
Employed	47.4	46.0	51.9
Unemployed	14.0	12.6	18.5
Student only	28.9	31.0	22.2
Student worker	8.8	10.3	3.7
Retired	0.9		3.7
Marital status			
Single	87.7	87.4	88.9
Married^a^	5.3	5.7	3.7
De facto union	5.3	5.7	3.7
Divorced	1.8	1.1	3.7
Gender identity (GI)			
Masculine	70.2	92.0	
Feminine	21.1		88.9
Non-binary	8.8	8.0	11.1
Gender expression (GE)			
Masculine	71.9	94.3	
Feminine	20.2		85.2
Androgenous	7.9	5.7	14.8
Gender-affirming hormone therapy (GAHT) duration			
1-2 years	38.6	41.4	29.6
2-5 years	40.4	40.2	40.7
≥ 5 years	21.1	18.4	29.6

Desired effects and side effects of GAHT

Table 2 presents participants’ ratings of GAHT’s physical effects after a period of at least one year of therapy.

**Table 2:** Desired physical changes rating (after at least one year of GAHT) Results are presented as median (P25 - P75) of the score attributed by the participants to the perception of changes development and satisfaction with them, for each mentioned alteration Abbreviations: GAHT, gender-affirming hormone therapy

	Perception of changes		Satisfaction with changes		
	n	Md (P25 - P75)	n		Md (P25 - P75)
Group M					
Amenorrhea	87	6.0 (5.0 - 6.0)	86	6.0 (6.0 - 6.0)	
Body hair increase	87	5.0 (4.0 - 6.0)	86	5.0 (3.0 - 6.0)	
Clitoris enlargement	86	6.0 (5.0 - 6.0)	86	6.0 (4.0 - 6.0)	
Facial hair increase	87	5.0 (3.0 - 6.0)	86	5.0 (3.0 - 6.0)	
Hips fat decrease	86	4.0 (3.0 - 5.0)	86	5.0 (3.0 - 6.0)	
Muscle mass increase	87	4.0 (3.0 - 5.0)	86	5.0 (3.0 - 6.0)	
Voice deepening	87	5.0 (4.0 - 6.0)	86	5.5 (4.0 - 6.0)	
Group F					
Body hair decrease	26	4.0 (2.0 - 6.0)	26	5.0 (2.0 - 6.0)	
Breast volume increase	27	4.0 (2.0 - 6.0)	27	4.0 (2.0 - 6.0)	
Facial hair decrease	26	4.0 (2.0 - 6.0)	26	4.5 (2.0 - 6.0)	
Hip fat increase	27	4.0 (2.0 - 5.0)	27	5.0 (2.0 - 6.0)	
Muscle mass decrease	27	4.0 (3.0 - 5.0)	27	4.0 (3.0 - 6.0)	
Semen production decrease	27	6.0 (2.0 - 6.0)	27	6.0 (4.0 - 6.0)	
Softer and less oily skin	27	5.0 (2.0 - 6.0)	27	5.0 (4.0 - 6.0)	
Spontaneous erections decrease	27	5.0 (4.0 - 6.0)	27	6.0 (5.0 - 6.0)	
Testicle size decrease	27	5.0 (2.0 - 6.0)	27	5.0 (2.0 - 6.0)	

In Group M, the changes evaluated with the best median score, concerning the participants’ perception of their appearance, were amenorrhea (6.0 (5.0 - 6.0)) and clitoris enlargement (6.0 (5.0 - 6.0)). The changes classified as most satisfactory were amenorrhea (6.0 (6.0 - 6.0)) and clitoris enlargement (6.0 (4.0 - 6.0)), followed by voice deepening (5.5 (4.0 - 6.0)).

As for Group F, regarding the perception of physical alterations, those evaluated with the best median score were the decrease in semen production (6.0 (2.0 - 6.0)), spontaneous erections (5.0 (4.0 - 6.0)), and testicle size (5.0 (2.0 - 6.0)), and softer/less oily skin (5.0 (2.0 - 6.0)). The physical changes rated as the most satisfactory were also the decrease in both spontaneous erections (6.0 (5.0 - 6.0)), and semen production (6.0 (4.0 - 6.0)). 

Regarding side effects, 89.7% of the participants from Group M experienced some adverse effect from GAHT, whilst 96.3% of the respondents from Group F reported at least one side effect.

Table 3 presents the participants’ stated side effects.

**Table 3:** Side effects of masculinizing and feminizing GAHT Results are presented as percentage (%) of participants who reported the mentioned side effect ^a ^Side effects Abbreviations: GAHT, gender-affirming hormone therapy

SE^a^ of masculinizing GAHT	Group M (n=87)	SE^a^ of feminizing GAHT	Group F (n=27)
Mood swings	56.3	Decreased libido	66.7
Acne	52.9	Mood swings	63.0
Alopecia	37.9	Exanthema	14.8
Pelvic/clitoral pain	23.0	Nausea/vomiting	14.8
Headaches	10.3	Headaches	11.1
Erythrocytosis	10.3	Dyslipidemia	7.4
Dyslipidemia	8.0	Hypertension	7.4
Hypertension	4.6	Venous thromboembolism	7.4
Exanthema	2.3	Stroke	3.7
Hepatic toxicity	2.3	Acute myocardial infarction	3.7
		Hyperprolactinemia	3.7

As shown, the most frequently stated side effects from participants in masculinizing therapy (Group M) were mood swings (56.3%), acne (52.9%), and alopecia (37.9%), while respondents who underwent feminizing therapy (Group F) mostly reported decreased libido (66.7%) and mood swings (63.0%).

Sociopsychological impact

Table 4 shows the sociopsychological impact of GAHT’s effects.

**Table 4:** Sociopsychological impact of GAHT’s physical changes Results are presented as median (P25-P75) of the score attributed by the participants to each variable pre- and post-therapy Abbreviations: GAHT, gender-affirming hormone therapy

Variable	Total			Group M			Group F		
	N	Md (P25 - P75)	p	n	Md (P25 - P75)	p	n	Md (P25 - P75)	p
Self-esteem									
Before	114	1.5 (1.0 - 2.0)	<0.001	87	1.0 (0.0 - 2.0)	<0.001	27	2.0 (1.0 - 2.0)	<0.001
After		5.0 (4.0 - 6.0)			5.0 (4.0 - 6.0)			5.0 (4.0 - 6.0)	
Body image satisfaction									
Before	114	1.0 (0.0 - 2.0)	<0.001	87	1.0 (0.0 - 2.0)	<0.001	27	1.0 (0.0 - 2.0)	<0.001
After		5.0 (4.0 - 5.0)			4.0 (3.0 - 5.0)			5.0 (4.0 - 6.0)	
Psychological well-being									
Before	114	3.0 (1.0 - 5.0)	<0.001	87	3.0 (1.0 - 5.0)	<0.001	27	3.0 (1.0 - 5.0)	0.406
After		5.0 (2.0 - 6.0)			5.0 (3.0 - 6.0)			3.0 (2.0 - 6.0)	
Social relations									
Before	113	3.0 (1.0 - 4.0)	<0.001	86	3.0 (1.0 - 4.0)	<0.001	27	3.0 (1.0 - 4.0)	0.003
After		5.0 (3.0 - 6.0)			5.0 (4.0 - 6.0)			5.0 (3.0 - 6.0)	
Family relations									
Before	114	3.0 (2.0 - 4.3)	<0.001	87	3.0 (2.0 - 5.0)	<0.001	27	3.0 (2.0 - 4.0)	0.019
After		4.0 (3.0 - 5.0)			4.0 (3.0 - 5.0)			5.0 (3.0 - 5.0)	

Participants retrospectively rated their self-esteem before and after GAHT with a median of 1.5 (M=1.0; F=2.0) and 5.0 (M|F=5.0) points, respectively. Of note, although the medians of the scores corresponding to pre-treatment self-esteem differed between Groups M and F, this difference was not statistically significant (p=0.417).

Median scores attributed to body image satisfaction were 1.0 (M|F=1.0) points before and 5.0 (M=4.0; F=5.0) points after therapy. Once again, while the medians of scores relative to post-therapy body image satisfaction attributed to each group were different, this difference was not statistically significant (p=0.206).

Respondents rated their psychological well-being with a median of 3.0 (M|F=3.0) points before and 5.0 (M=5.0; F=3.0) points after GAHT. Even though the medians of the scores attributed to post-treatment psychological well-being differed between Groups M and F, this difference was not statistically significant (p=0.180).

Participants also rated their social relations before and after therapy with 3.0 (M|F=3.0) and 5.0 (M|F=5.0) points, respectively. 

Lastly, their family relations were evaluated with a median of 3.0 (M|F=3.0) points pre-treatment and 4.0 (M=4.0; F=5.0) points post-treatment. Nonetheless, the difference between the post-treatment medians of scores of both groups was not statistically significant (p=0.908).

All analyzed variables had a statistically significant improvement (p<0.001) with GAHT, both in the total sample and in Group M. In Group F, all variables improved significantly, except for psychological well-being (p=0.406).

Satisfaction

These participants (who underwent at least one year of uninterrupted GAHT) rated their overall satisfaction with treatment results with a median of 5.0 (M|F=5.0) points and their satisfaction with received medical follow-up with a median of 4.5 (M=4.0; F=5.0) points. The difference between the medians of the scores attributed to the received medical follow-up by Groups M and F was not statistically significant (p=0.681).

## Discussion

The 2015 U.S. Transgender Survey, conducted by the National Center for Transgender Equality, revealed that among transgender individuals using GAHT, 39% reported using testosterone, while 64% reported using estrogen and/or progesterone, evidencing a much greater number of people seeking feminizing treatments [8]. In contrast, our study demonstrated an inverse relationship, with a larger proportion of individuals seeking masculinizing hormone treatments. This correlation was likewise observed in two additional studies conducted on the transgender population in Portugal, suggesting that this might be characteristic of the Portuguese context [4,9]. While there is no current explanation for this, we suggest that cultural and societal factors, as well as differences in healthcare access and availability of hormone therapy options, may contribute to the observed disparities. Sample size, recruitment methods, and study design are important considerations that may also influence these findings. Further research is needed to better understand the underlying factors that may explain these differences. 

Our study results were consistent with other international studies that have shown the significant physical changes induced by GAHT [10-18]. Our study reinforces the findings of others that side effects are mostly mild, with acne and libido loss being among the most frequent in transgender individuals receiving masculinizing or feminizing hormone therapy, respectively, and that severe adverse alterations, such as erythrocytosis, venous thromboembolism or even important hepatic toxicity seem to be rare [16-19].

Previous research has demonstrated the positive impact of GAHT on body image and self-esteem in transgender individuals, with findings similar to ours. GAHT can improve gender-body conformation, meaning that transgender individuals who undergo GAHT tend to experience a greater sense of alignment between their gender identity and their physical appearance [13,20-23]. This, in turn, can reduce body image dissatisfaction and increase satisfaction with appearance, as well as overall self-esteem [13,20-23]. These positive effects on body image and self-esteem can have significant implications for mental health, as body dissatisfaction and low self-esteem are risk factors for depression and anxiety [13,20-23]. 

The results here presented, similarly to the ones of other studies, also demonstrated improvements in psychological well-being and reduced risk of suicidal ideation following GAHT. A probable explanation for this is that making the body characteristics more congruent with the experienced gender reduces dysphoria and even potentially decreases marginalization, allowing better results in mental health, which considerably diminishes the risk of suicide attempt [24-26]. Psychological well-being did not improve in a statistically significant way in Group F, possibly because of the greater social stigma that trans women face, in comparison to trans men, even after transition. A study from Verbeek et al. (2020) showed that trans women deal with higher social stigma, not only because of misogyny itself but also for being perceived as a threat to cisgender women [27].

Our study also revealed an improvement in social and family relations after hormone therapy, as suggested by other researchers. Although individual experiences may vary, since not all transgender individuals have supportive families and peers, and some may experience rejection or hostility, several studies have found that gender-affirming treatment can result in positive changes in relationships [20,21,28,29]. A study by Gorin-Lazard et al. (2017) found that transgender individuals who underwent hormone therapy reported improvements in their family relationships, including increased acceptance and support from family members [20]. This may occur because gender-affirming treatments seem effective in reducing gender dysphoria and improving overall well-being in transgender people, which can have positive effects on relationships with family members and other social connections [20,21,28,29].

Other studies have also reported significant improvements in the quality of life of transgender individuals who undergo GAHT. Our study similarly found that individuals who received GAHT reported a high level of satisfaction with the results, which suggests that hormone therapy can have a positive impact on the overall well-being of transgender individuals. The aforementioned improvements in various areas of transgender individuals' lives may collectively contribute to the substantial enhancements in their reported quality of life [20,30].

All these findings are in line with the growing body of evidence that supports the use of GAHT as a crucial component of gender-affirming care.

Strengths and limitations

The current study has several notable strengths that contribute to the field of research. Firstly, the study achieved a high participant count relative to the expected size of the target population, indicating a strong level of engagement and interest from individuals within the studied population. Secondly, the study was able to acquire data regarding variables that had not been previously explored in related investigations, providing new insights into the topic under investigation. Additionally, the study represents one of the few investigations of its kind conducted in Portugal, emphasizing the significance of its contribution to the field. Together, these strengths highlight the value of the current study and provide an additional foundation for future research in this area.

Nonetheless, several potential limitations should be taken into account when interpreting the results. Firstly, differences between the number and characteristics of the participants from each group, as well as a small sample size for some variables, participant selection procedures and limited data comparison and analysis may have influenced the study's findings. In addition, the individualized nature of GAHT regimens, different treatment duration for each participant, the uncertainty of medication adherence, and the inclusion of participants from various countries with potential interpopulational and biological differences should also be acknowledged. Furthermore, the cohort mainly consisting of participants from Portugal limits the generalization of the results. Moreover, due to the retrospective nature of the study and the lack of a validated questionnaire, participants' evaluation of pre-treatment variables may have been less accurate. Finally, the potential for bias in participants' responses due to the "sunk cost fallacy," along with other factors such as concurrent treatment or personal life events that may have influenced the results, were not controlled for in the study. As a result, caution should be exercised when generalizing the findings to other populations or settings.

Gender dysphoria is a recently acknowledged issue, and it has only recently gained recognition in the field of medicine. There is still a dearth of large-scale, prospective studies that are necessary to optimize its management and to provide individuals who identify as transgender with the best possible treatment options. To address this gap in knowledge, it is critical to undertake research that employs more standardized methods of data collection and that follows clearly delineated treatment protocols.

## Conclusions

This study provides further evidence that GAHT is effective, and several expected satisfactory changes can be observed after at least one uninterrupted year of hormonal therapy. The adverse effects reported were also mostly non-severe and non-life-threatening. For transgender people who seek transition, GAHT is consistently associated with improvements in self-esteem, body image satisfaction, psychological wellbeing, and social and family relations. Our study also revealed that there is general satisfaction with both therapy results and medical follow-up.

This study brings new evidence to physicians that GAHT seems to be important to increase the physical, psychological and social health of transgender people seeking medical transition, probably associated with the improvement in the quality of life verified in other studies.
